# Dexrazoxane prevents vascular toxicity in doxorubicin-treated mice

**DOI:** 10.1186/s40959-024-00270-w

**Published:** 2024-10-04

**Authors:** Dustin N. Krüger, Matthias Bosman, Emeline M. Van Craenenbroeck, Guido R. Y. De Meyer, Constantijn Franssen, Pieter-Jan Guns

**Affiliations:** 1 Laboratory of Physiopharmacology, Faculty of Medicine and Health Sciences, Faculty of Pharmaceutical, Biomedical and Veterinary Sciences, Campus Drie Eiken, University of Antwerp, Universiteitsplein 1, Antwerp, B-2610 Belgium; 2 Research Group Cardiovascular Diseases, University of Antwerp, Antwerp, B-2610 Belgium; 3 Department of Cardiology, Antwerp University Hospital (UZA), Drie Eikenstraat 655, Edegem, B-2650 Belgium

**Keywords:** Doxorubicin, Dexrazoxane, Vascular toxicity, Endothelial cell dysfunction, SERPINA3

## Abstract

**Background:**

Doxorubicin (DOX) is used for breast cancer and lymphoma, but can cause cardiotoxicity, arterial stiffness, and endothelial dysfunction. We recently reported SERPINA3N as biomarker of cardiovascular toxicity in patients and mice. Dexrazoxane (DEXRA) is an FDA-approved drug that prevents DOX-induced cardiac toxicity in high-risk patients. However, the effect of DEXRA on vascular dysfunction during DOX treatment has not been documented. Therefore, here we investigated whether DEXRA protects against DOX-induced arterial stiffness, endothelial dysfunction, and SERPINA3N upregulation in tissue and plasma from mice.

**Methods:**

Male C57BL6/J mice were treated with DOX (4 mg/kg), DEXRA (40 mg/kg), a combination (DEXRA + DOX), or VEHICLE (0.9% NaCl) weekly i.p. for 6 weeks (*n* = 8 per group). Cardiovascular function was measured in vivo by ultrasound imaging at baseline, weeks 2 and 6. Vascular reactivity was analyzed ex vivo in the thoracic aorta at week 6 and molecular analysis was performed.

**Results:**

DEXRA prevented left ventricular ejection fraction decline by DOX (DEXRA + DOX: 62 ± 2% vs DOX: 51 ± 2%). Moreover, DEXRA prevented the increase in pulse wave velocity by DOX (DEXRA + DOX: 2.1 ± 0.2 m/s vs DOX: 4.5 ± 0.3 m/s) and preserved endothelium-dependent relaxation (DEXRA + DOX: 82 ± 3% vs DOX: 62 ± 3%). In contrast to DOX-treated mice, SERPINA3N did not increase in the DEXRA + DOX group.

**Conclusion:**

Our results not only confirm the cardioprotective effects of DEXRA against DOX-induced cardiotoxicity but also add preservation of vascular endothelial cell function as an important mechanism. Moreover, the study demonstrates the potential of SERPINA3N as a biomarker for monitoring cardiovascular complications of DOX in high-risk patients.

**Supplementary Information:**

The online version contains supplementary material available at 10.1186/s40959-024-00270-w.

## Background

Anthracyclines remain a therapeutic cornerstone for several malignancies, such as breast cancer and lymphomas. However, the risk of cardiotoxicity from anthracyclines is a major concern [1]. Moreover, anthracyclines also cause vascular toxicity, such as endothelial cell (EC) dysfunction and arterial stiffness [2–4]. EC dysfunction has been shown to be an early hallmark of anthracycline-induced toxicity in breast cancer patients [5]. We have previously shown in mice that doxorubicin (DOX), the most commonly used anthracycline, induces EC dysfunction within a few hours (early phase) after a single injection of DOX [6] and that this persists for multiple days (late phase) [7]. Furthermore, vascular smooth muscle cells (VSMC) function was affected, causing early-phase hypocontractility and late-phase hypercontractility [2]. Several reports have shown increased pulse wave velocity (PWV), a marker of arterial stiffness, in anthracycline-treated patients (reviewed in [4]), which we could link to EC dysfunction in DOX-treated mice [2].

Both EC dysfunction and arterial stiffness have been associated with arterial aging [8], suggesting that anthracyclines cause accelerated aging. Likewise, epidemiological data indicate that cancer survivors treated with anthracyclines have a higher risk of developing hypertension and coronary artery disease later in life, with long-term vascular effects being most pronounced in pediatric cancer patients [9, 10]. Although the vascular toxicity of anthracyclines has received much less attention than cardiotoxicity, it represents an interesting target for reducing long-term cardiovascular complications of anthracyclines.

While many cardioprotective strategies have been evaluated in experimental studies, dexrazoxane (DEXRA) is the only available option to prevent cardiotoxicity in patients induced by anthracyclines [1]. DEXRA has been approved by the FDA as a preventive strategy in high-risk patients receiving cumulative doses of DOX > 300 mg/m^2^ and continuing to receive DOX to maintain tumor control [1, 11–13]. Several mechanisms of action have been proposed for DEXRA`s protective effect. First, DEXRA interacts with TOPOISOMERASE-2β (TOP-2β), preventing DOX-induced DNA damage [14]. Second, DEXRA is an ethylenediaminetetraacetic acid analogue, and as such protects against ROS by chelating iron and scavenging free radicals [14]. Indeed, DOX induces iron-mediated oxidative stress and ferroptosis, thereby damaging the cardiovascular system [15, 16].

In summary, while the protective effects of DEXRA on the myocardium have been established, the potential benefits to the vasculature and, more specifically, the endothelium are unknown. We previously observed no protective effects of DEXRA on EC function in an acute, short-term (16 h) mouse model of DOX-induced cardiovascular toxicity (CVT) [7]. Therefore, a chronic, long-term (6 weeks) DOX protocol was applied in the current study to investigate whether DEXRA protects against vascular toxicity. In addition, we investigated the effect of DEXRA on SERPINA3N (human ortholog SERPINA3) expression, which we have previously proposed as a promising marker of CVT in both DOX-treated patients and mice [2].

## Materials & methods

### Animals and ethical approval

Male C57BL/6 J mice (age: 10 weeks; body weight: 24–30 g; Charles River) were housed in the animal facility of the University of Antwerp in standard cages with 12–12 h light–dark cycles with access to water ad libitum and regular chow. All experiments were approved by the Animal Ethics Committee of the University of Antwerp (file 2021–19) and conformed with the ARRIVE guidelines, in accordance with Directive 2010/63/EU and with the Belgian Royal Decree of 2013. Welfare of animals was assessed daily by the animal caretakers and the principal researcher based on the scoring system of the Functional Observation Battery. Criteria for humane euthanasia were as follows: Animals displaying obvious signs of pain based on the scoring table, meeting specific score thresholds, or exhibiting significant weight loss (> 20%).

### Study design

The study included 4 treatment groups: DOX (Adriamycin®, 2 mg/mL, Pfizer, Belgium) (4 mg/kg), a combination of DOX (4 mg/kg) and DEXRA (Tocris Bio-Techne, Dublin, Ireland) (40 mg/kg), DEXRA (40 mg/kg) or VEHICLE (10 mL/kg of a 0.9% NaCl solution; B. Braun, Belgium). Compounds were administered intraperitoneally weekly to the 4 randomly divided groups of C57BL/6 J mice (*n* = 8 per group). DEXRA dose of 40 mg/kg was chosen as the clinically suggested dosage ratio of DEXRA to DOX is 10 to 1. DEXRA was administered 30 min before DOX, as performed in patients. The animals were treated for 6 weeks (cumulative dose DOX 24 mg/kg, DEXRA 240 mg/kg). In weeks 2 (14 days after beginning of treatment, right before 3rd injection) and 6 (2 days after last injection) cardiac and vascular function were measured using ultrasound. 2–3 days after the last ultrasound imaging, mice were sacrificed to assess ex vivo vascular reactivity and tissue and plasma were procured. Isoflurane was used as sedative during ultrasound imaging and this is known to induce vasodilation [17]. To avoid possible interference of isoflurane, an interval of 2–3 days was respected between imaging procedures and ex vivo vascular reactivity experiments. For simplification purposes, the timepoint for the final ultrasound session, vascular reactivity measurements and molecular analysis will be abbreviated as week 6.

### Ultrasound imaging

Mice were first anaesthetized using isoflurane (induction concentration of 3% v/v) and were maintained sedated at 1–2% v/v. They were then placed on the Vevo F2 LAZR-X imaging station (Visual Sonics—FUJIFILM), which is heated to stabilize the body temperature of the animals at 37 ± 1 °C and enable the recording of physiological parameters. Heart rate and respiration were constantly monitored and maintained at approximately 400–500 beats per minute and 100 breaths per minute, respectively.

Echocardiographic measurements were conducted using a 57 MHz transducer (Visual Sonics) and images were recorded from different windows. Left ventricular function and dimensions were measured as indices of systolic function (M-mode). PWV was measured in the abdominal descending aorta. Additionally, diastolic function was assessed using the mitral valve E/A ratio (MV E/A) and the E/E’ ratio (MV E/E’). These measurements were performed on at least three heartbeats from each view and then averaged.

###  Evaluation of ex vivo vascular reactivity

Mice were euthanized 4–5 days after the last injection (week 6) and thoracic descending aortic segments were procured and mounted at a preload of 20 mN, and the experimental protocol was started 50 min thereafter to allow optimal stabilization. Vascular smooth muscle cell (VSMC) contraction was evaluated by adding a single dose of phenylephrine (Sigma-Aldrich Overijse, Belgium) (2 µM), an α1-adrenergic receptor agonist, for 15 min. Next, endothelium-dependent vasodilation was investigated by addition of cumulative concentrations of acetylcholine (Sigma-Aldrich Overijse, Belgium) (3 nM–10 μM), a muscarinic receptor agonist. After washing steps to remove phenylephrine and acetylcholine, phenylephrine -stimulated contraction was repeated, and once stable, Nω-nitro-L-arginine methyl ester (Sigma-Aldrich Overijse, Belgium) (L-NAME; 300 µM), an inhibitor of endothelial nitric oxide synthase (eNOS), was subsequently added. After 20 min, cumulative concentrations of the exogenous nitric oxide (NO)-donor diethylamine NONOate (DEANO, Sigma-Aldrich Overijse, Belgium) (0.3 nM–10 μM) were added to the organ bath to evaluate endothelium-independent vasodilation of VSMCs through the cGMP-mediated pathway.

### RNA isolation and cDNA synthesis

RNA was isolated and purified from two 2 mm thoracic aortic segments and cardiac tissue (apex) using the RNeasy® Micro Kit (QIAGEN) or ISOLATE II RNeasy® Mini Kit (QIAGEN), respectively, according to the manufacturer’s instructions. RNA purity and concentration were measured with the NanoDrop® ND-1000 spectrophotometer. Next, reverse transcription was performed using the TaqMan™ Reverse Transcription Reagents kit (Thermo Fisher Scientific; Invitrogen), according to the manufacturer’s instructions. Random hexamers (2.5 µM), provided with this kit, were used as primers.

### Quantitative Real-time PCR

Then RT-qPCR was performed with the TaqMan™ Fast Advanced Master Mix (Thermo Fisher Scientific) and the TaqMan™-probes, Mm01254822_m1 for *Ferroportin-1*, Mm00776439_m1 for *Serpina3n*, Mm00449032_g1 for *β-actin* and Mm99999915_g1 for *Gapdh* (Thermo Fisher Scientific) in a QuantStudio™ 3 Real-Time PCR system (Applied Biosystems™). PCR was performed according to the manufacturer.

### Western blotting

Cardiac and thoracic aorta samples were used for all western blotting experiments. Cardiac tissue was homogenized in RIPA lysis buffer (abcam), supplemented with 1 tablet of protease inhibitor (cOmplete Mini, Roche) and 1 tablet of phosphatase inhibitor PhosSTOP (EASYpack, Roche), using the Precellys® 24. Samples were diluted in Laemmli buffer (Bio-Rad) containing 5% β-mercaptoethanol (Sigma-Aldrich) to reach a final concentration of 1 µg/µL. For thoracic aorta samples, one 2 mm segment for each condition was directly lysed in 50 µL Laemmli buffer containing 5% β-mercaptoethanol. All samples were subsequently heat-denatured for 5 min at 100 °C. Next, samples were loaded on Bolt 4–12% Bis–Tris gels (Invitrogen) and after electrophoresis transferred to Immobilon-FL PVDF membranes (Merck). Afterwards, total protein stain was performed using Revert™ 700 Total Protein Stain (Li-COR, Biosciences). After blocking (1 h, Odyssey Li − COR blocking buffer (Li-COR Biosciences)), membranes were probed with primary antibodies, diluted in Odyssey Li − COR blocking buffer, overnight at 4 °C. The following primary antibodies were used: mouse anti-TOPOISOMERASE-2β (1:1000, TOP-2β, ab125297; abcam), rabbit anti-eNOS (1:1000, phsopho Ser-1177, ab215717; abcam) and mouse anti-eNOS (1:1000, ab76198; abcam). The next day, membranes were incubated with IRDye-labeled secondary goat anti-rabbit IgG926-68,171 (Li − COR Biosciences) and goat anti-mouse IgG926-32,210 (Li − COR Biosciences) for 1 h at room temperature. Membranes were visualized with an Odyssey SA infrared imaging system (Li − COR, Biosciences). Signal of the protein of interest was corrected for the total protein stain (loading control).

### Histology

After dissection, cardiac tissues were washed in Krebs–Ringer solution, immediately submerged in 4% formaldehyde, buffered pH = 7 (Merck, Overijse, Belgium) and stored at room temperature for 24 h. The next day segments were dehydrogenized in 60% isopropanol for 24 h at 4 °C and embedded in paraffin. On the day of staining for immunohistochemistry (IHC), segments were transversely cut into 10 µm segments. A primary antibody Anti- γH2A.X (phsopho S139) (1:500; ab 11,174 Abcam, Belgium) was used to investigate DNA damage in cardiac tissue. Heat using a rabbit anti-goat biotinylated secondary antibody (1:200; Vector Laboratories, Burlingame, USA), cardiac tissues were stained with 3,3'-diaminobenzidine (Sigma Aldrich, Belgium). The nuclei were stained for 30 s in hematoxylin.

### ELISA

To investigate circulating SERPINA3N levels in the mice an ELISA Kit from Aviva Systems Biology (OKEH03592, California, USA) was used. Blood of the animals was collected on the day of dissection retroorbital in an Eppendorf tube containing 20 µL of 10% EDTA (pH 7.6, Sigma Aldrich, Belgium). The Kit was performed according to the manufacturer's instructions. The plasma samples from the animals were diluted 1 in 10 before the start of the Kit. Before the conduction of the Kit, all samples and reagents were kept at room temperature for 60 min.

### Statistical analysis

All results are expressed as the mean ± standard error of the mean (SEM). Statistical analyses were performed using GraphPad Software (Prism 10—Version 10.0.1; GraphPad, California, United States of America). A *p*-value < 0.05 was considered statistically significant. The following statistical tests were performed, based on normal distribution of the values using Shapiro–Wilk test: Two-Way ANOVA and a Tukey correction for left ventricular ejection fraction (LVEF), PWV, organ weight, and vascular reactivity. Kruskal-Walis test followed by Dunn`s multiple comparisons for western blot, qPCR, and other echocardiographic parameters.

## Results

### DEXRA prevents CVT in mice

**Fig. 1:**
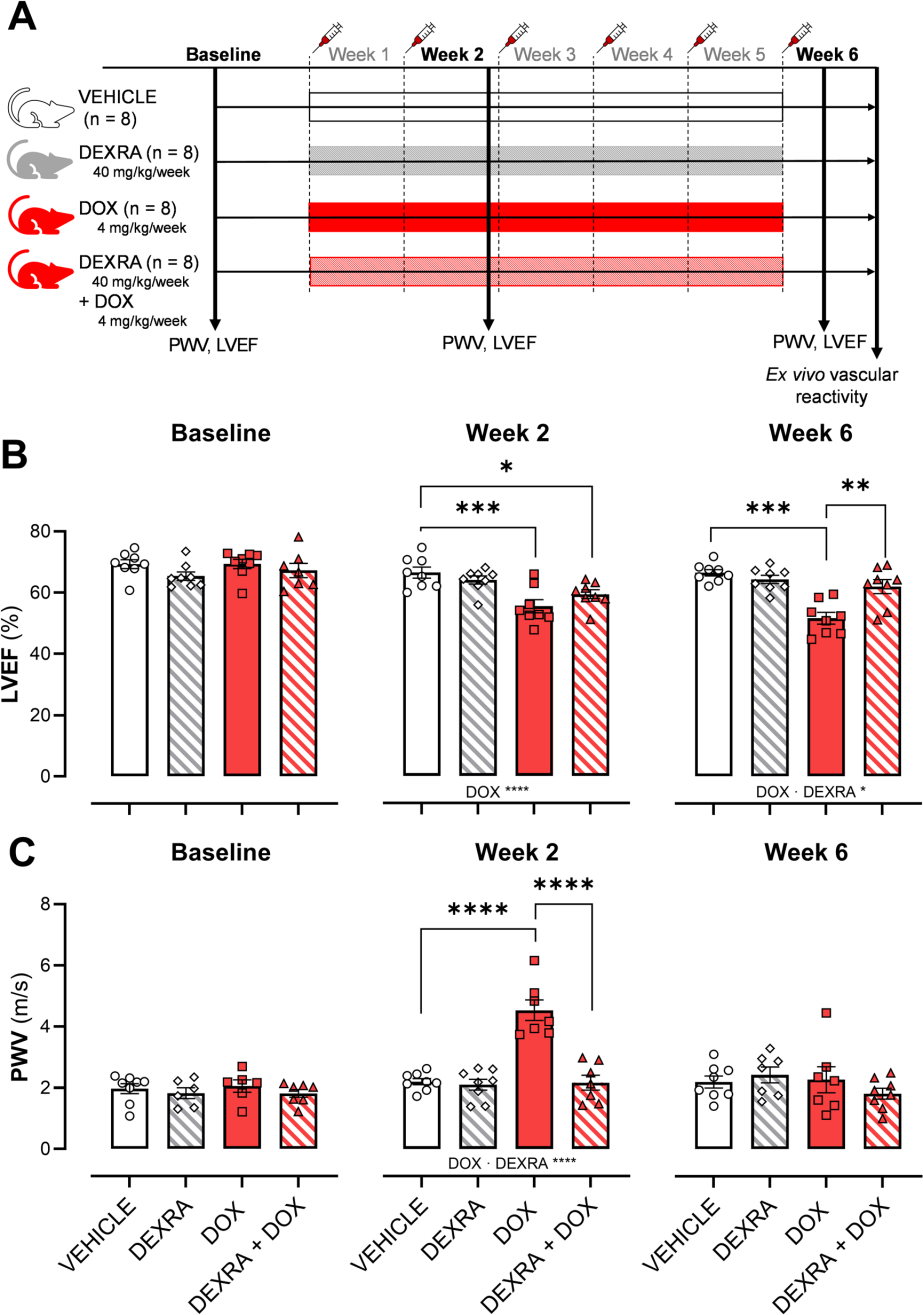
DEXRA prevents DOX-induced cardiotoxicity and arterial stiffness in the abdominal aorta. **A** Experimental setup. Mice were treated for 6 weeks with either DOX, DEXRA, a combination of both, or VEHICLE. Echocardiographic measurements of week 6 were performed 2 days after their final injection. **B** LVEF at baseline and week, 2 and 6 in all groups. **C** Assessment of PWV in the abdominal aorta in vivo at baseline and weeks, 2 and 6. LVEF, left ventricular ejection fraction; PWV, pulse wave velocity. Data is shown as mean ± SEM. Two-way ANOVA and a Tukey correction. For each cohort: *n* = 8. *, **, ***, ****, *p* < 0.05, 0.01, 0.001, 0.0001

Figure 1A includes the study design and treatment regime for the animals as described in the methods section. All mice survived the cumulative treatment.

The diagnosis of DOX-induced CVT is typically based on the reduction in LVEF [1]. Starting from week 2 and also measured at week 6, DOX-treated mice exhibited consistently diminished LVEF (*P* = 0.0005) compared to other groups (Fig. 1B). Conversely, mice receiving a combination of DEXRA and DOX tended to show less reduction in LVEF from baseline (ΔLVEF_DEXRA+DOX_: -11 ± 1% vs ΔLVEF_DOX:_ -17 ± 2%) at week 2. While at week 6 DEXRA-treated mice, did not show a reduction in LVEF (DEXRA + DOX: 62 ± 2% vs DOX: 51 ± 2%, *P* = 0.011). Further, DOX-treated mice showed reduced fractional shortening at weeks 2 (*P* = 0.006) and 6 (*P* = 0.003), while DEXRA was protective at week 6 (Supplementary Table 1). Additionally, DOX reduced stroke volume at weeks 2 (*P* = 0.009) and 6 (*P* = 0.0004). LV anterior wall thickness decreased in DOX-treated mice (*P* = 0.0319) at week 6. Further LV internal diameter was reduced in DOX-treated mice at weeks 2 (*P* = 0.047) and 6 (*P* = 0.0055) (Supplementary Table 1). Mice treated with DOX alone (*P* = 0.0022) or in combination with DEXRA (*P* = 0.017) had significant weight loss (Supplementary Fig. 1A), while DEXRA alone had no effects on body weight. DOX-treated mice displayed increased lung weights relative to body weight after 6 weeks (*P* = 0.016) (Supplementary Fig. 1C), while heart weight remained unaffected by any treatment intervention. Treating with DEXRA diminished the effect of DOX on lung weight.

DOX increased arterial stiffness illustrated by higher PWV in the abdominal aorta after 2 weeks of treatment (cumulative 8 mg/kg, *P* < 0.0001) (Fig. 1C). Additionally, DEXRA pre-treatment prevented elevation of PWV (*P* < 0.0001) (Fig. 1C). After 6 weeks, PWV in DOX-treated (cumulative 24 mg/kg) mice showed no significant difference compared to the other groups in line with previous reports from our group [2].

### DEXRA prevents DOX-induced EC dysfunction

**Fig. 2:**
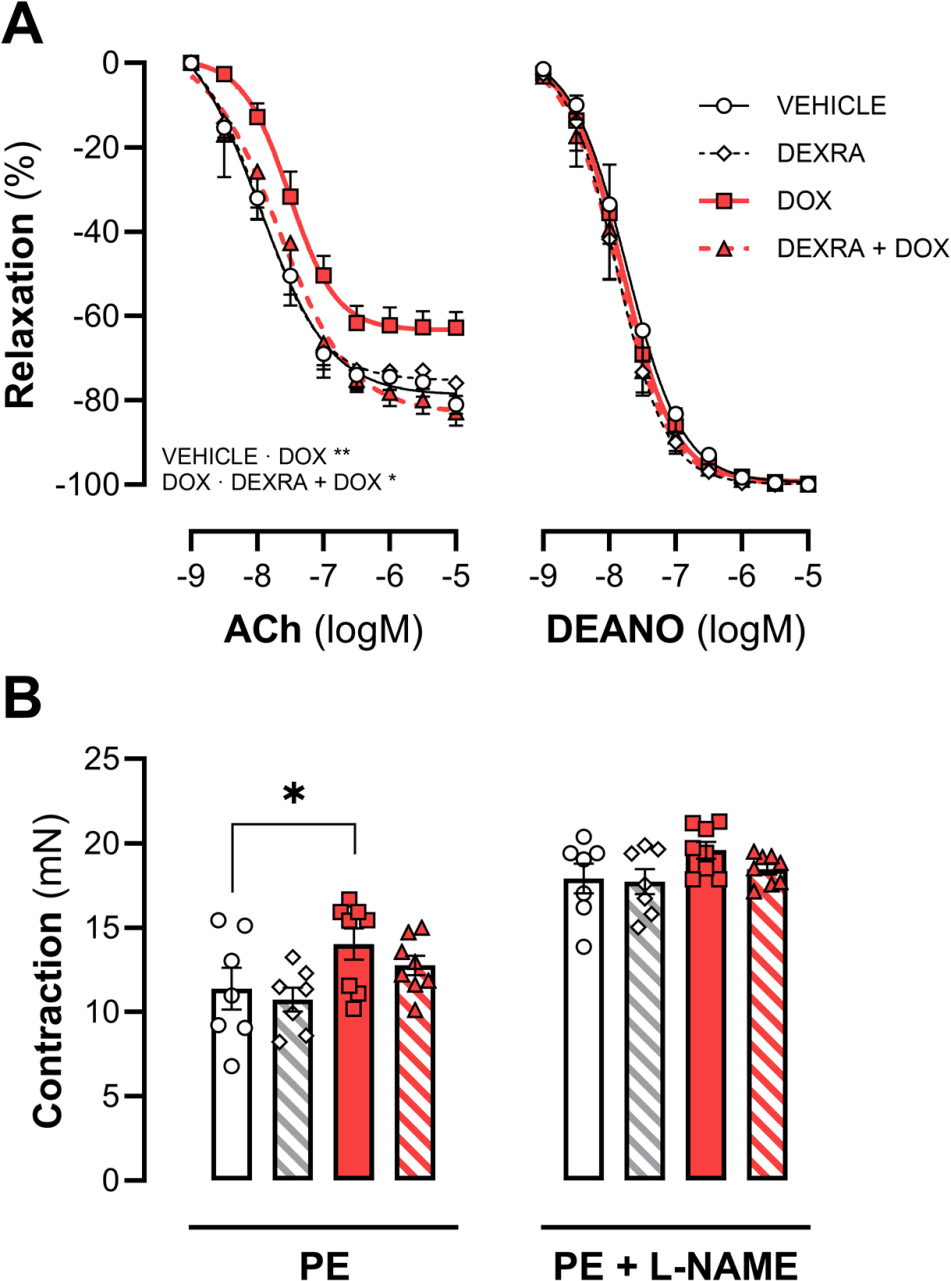
Vascular reactivity in thoracic descending aorta. DEXRA combined with DOX does not affect endothelial-dependent vasorelaxation **A** Vascular reactivity, measured ex vivo*.* Vasodilatation was assessed endothelial-dependent and -independent with ACh and DEANO, respectively. **B** Vasocontraction and basal NO were measured with PE and PE + L-NAME. At week 6 a cumulative dose of 24 mg/kg DOX and 240 mg/kg DEXRA was used. Data shows mean ± SEM. ACh, acetylcholine; DEANO, diethylamine NONOate; L-NAME, Nω-nitro-L-arginine methyl ester; PE, phenylephrine. **A** & **B**: Two-way ANOVA, *, **, *p* < 0.05, *p* < 0.01

Vascular reactivity of the thoracic descending aorta was assessed ex vivo after 6 weeks. Endothelium-dependent vasorelaxation, stimulated by acetylcholine, was compromised in the DOX-treated group (*P* = 0.0027) (Fig. 2A). Pretreatment with DEXRA restored EC dysfunction induced by DOX (*P* = 0.013). In the absence of DOX, DEXRA did not affect acetylcholine-induced vasorelaxation. Alternatively, neither DOX nor the combination of DOX and DEXRA affected endothelium-independent vasorelaxation induced by DEANO. Notably, phenylephrine-induced contraction was increased in the DOX group (*P* = 0.0465) relative to the VEHICLE group (Fig. 2B) and DEXRA showed a tendency to lower contractions. However, in the absence of NO (after inhibition of eNOS by 300 µM L-NAME) the maximum contraction induced by phenylephrine (2 µM), was similar between the groups.

### DEXRA prevents SERPINA3N upregulation after DOX

**Fig. 3:**
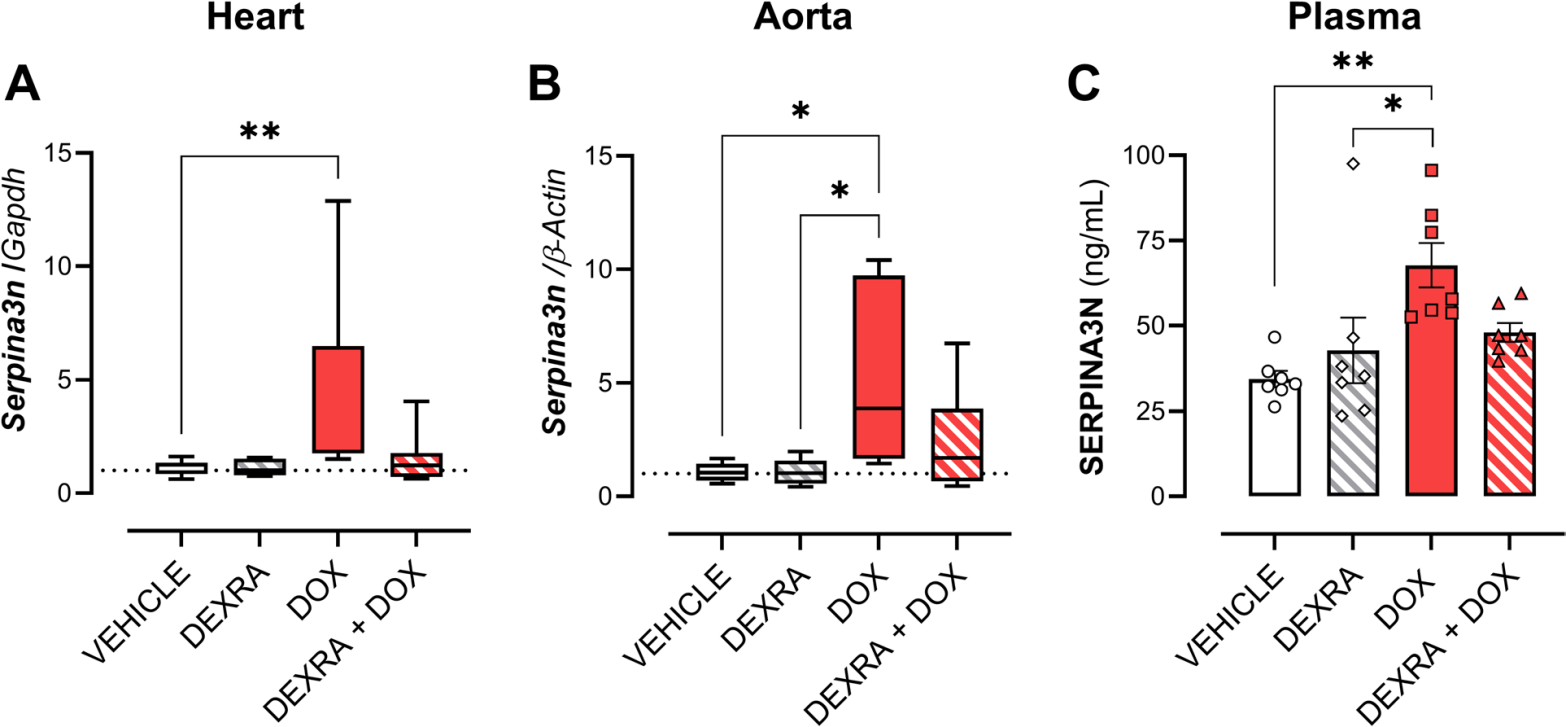
SERPINA3N expression and plasma circulation in mice after DOX treatment. DOX increases mRNA of *Serpina3n* as well as circulating SERPINA3N levels after 6 weeks of treatment. **A** *Serpina3n* expression in cardiac tissue. **B** *Serpina3n* expression in aortic tissue. **C** Plasma concentration of SERPINA3N. At week 6 a cumulative dose of 24 mg/kg DOX and 240 mg/kg DEXRA was used. Data shows mean ± SEM. A-C Two-Way ANOVA and a Tukey correction. For each cohort: *n* = 8. *, **, *p* < 0.05, 0.01

Gene expression of *Serpina3n* in cardiac (*P* = 0.016) and aortic tissue (*P* = 0.002) was increased in DOX-treated animals, but not when mice were pretreated with DEXRA (Fig. 3A&B). Likewise, circulating SERPINA3N levels were increased in DOX-treated mice (*P* = 0.002), but not in mice receiving a combination of DEXRA and DOX. DEXRA alone did not affect the plasma SERPINA3N levels (Fig. 3C).

**Fig. 4:**
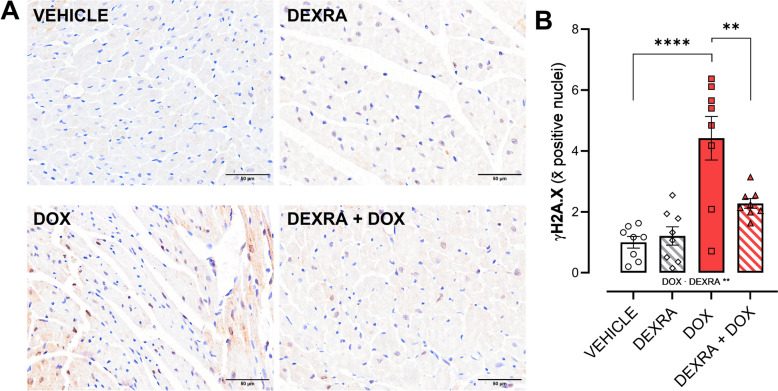
DOX-induced DNA damage in cardiac tissue after 6 weeks of treatment. **A** Representative image of γ-H2AX stained cardiac tissue. **B** Quantification of positively stained nuclei. A combination treatment of DOX and DEXRA reduced the amount of γH2A.X. Graph shows average positive nuclei per image. At week 6 a cumulative dose of 24 mg/kg DOX and 240 mg/kg DEXRA was used. Data shows mean ± SEM. The scale bar is 50 µm. B; Two-Way ANOVA and a Tukey correction. For each cohort: *n* = 8. **, ****, *p* < 0.01, 0.0001

Representative images of γ-H2AX stained myocardial tissue sections are shown in Fig. 4A. In cardiac tissue, more γ-H2AX positive nuclei were measured in DOX-only treated animals (*P* < 0.0001), compared to the other groups. The combined treatment resulted in fewer positive nuclei (*P* = 0.004) (Fig. 4B).

Further, given previous reports pointing towards depletion of TOP-2β by DEXRA, expression of TOP-2β in the heart and aorta was investigated. In brief, TOP-2β, protein levels were affected neither in the heart nor aorta (Supplementary Fig. 2A&D). Further, eNOS and phosphorylated eNOS (Ser-1177) were quantified by western blot of aorta samples, but no differences were detected among groups (Supplementary Fig. 3B&C). Additionally, the iron transporter *Ferroportin-1* expression was determined by Rt-qPCR as a possible marker of ferroptosis induced by DOX. DOX treatment augmented the expression of *Ferroportin-1* in cardiac tissue (*P* = 0.019) which was partly suppressed by DEXRA (although not statistically significant). The expression of *Ferroportin-1* in aortic segments was more variable rendering these results rather inconclusive (Supplementary Fig. 4A&B).

## Discussion

The present study demonstrates for the first time that DEXRA prevents DOX-induced vascular toxicity and confirms the well-documented cardioprotective effects of DEXRA. While DOX-induced PWV, a marker of arterial stiffness, increased, this detrimental response was not observed when DEXRA preceded DOX. Similarly, ex vivo endothelial-dependent relaxations were impaired by DOX but fully restored when mice were pretreated with DEXRA. Moreover, DOX caused a consistent rise in SERPINA3N expression in aorta and myocardium, as well as in plasma, which was not present in mice treated with a combination of DEXRA and DOX.

### Vascular toxicity prevented by DEXRA

Arterial stiffening induced by anthracyclines has been observed in cancer patients in the early phase (3 months) of anthracyclines therapy [18–20]. In the present study, DEXRA prevented an increase in PWV (at week 2) in mice, suggesting a protective effect of DEXRA beyond the heart. Interestingly, the initial increase of PWV at week 2 was consistent across studies [2, 6], but this phenomenon disappeared at week 6. A similar observation was made in female breast cancer patients, where increased PWV returned to baseline when treatment was completed [18]. The mechanism of the normalization of PWV is not precisely known, but previous studies in mice have not observed vascular remodeling [2]. An alternative explanation could be the reduction in systolic function induced by DOX. Indeed, human data have highlighted a negative correlation between LVEF and PWV in heart failure patients [21]. Collectively, these data showed that PWV is dynamic, time-dependent, and less suitable as functional marker in patients at risk for CVT.

EC dysfunction occurs early after anthracycline administration in humans [22] but has been underexplored. In mice, EC dysfunction is present within hours (16 h) after a single DOX injection [3]. In a previous study, we failed to demonstrate protective effect of DEXRA in such acute setting [7]. In contrast, the current study utilized a chronic 6-week repeated DOX injection protocol, which did show protective effects of DEXRA on EC function. This underlines the importance of timing in vascular effects of DOX. The initial phase is characterized by EC dysfunction and VSMC hypocontraction [3, 6, 7]. In contrast, during the later phase, VSMC hypercontraction is observed, as well as a consistent EC dysfunction. During this later phase (i.e. after a few days), DOX concentrations are likely lower due to washout [2]. By this time, partial recovery of VSMCs may occur, potentially supported by interventions such as DEXRA, which may aid in the recovery process. While DEXRA in the current study tends to prevent hypercontractility of the VSMC, DEXRA did previously not prevent hypocontractility in the early phase [7]. It is speculative that, in the early phase DOX causes acute cellular damage or disrupt signaling pathways, such as calcium handling [23, 24], which DEXRA is unable to counteract effectively. Over the longer term, chronic exposure to DOX could result in compensatory mechanisms or adaptive changes within VSMCs that manifest as hypercontractility, a condition where DEXRA's protective effects are more pronounced. In part, hypercontractility of VSMC may relate to impaired EC function with reduced availability of NO, a potent vasodilator [25, 26].

### Mechanism of DEXRA preservation of EC function

Several mechanisms of EC dysfunction induced by DOX have been proposed, such as direct cellular damage due to ROS and DNA double-strain breaks [27], limited bioavailability of NO in the presence of ROS or interactions of DOX with eNOS [28–30]. DEXRA has direct antioxidative activity [31–33] Further, the hydrolyzed metabolite of DEXRA (i.e., ADR-925) is a potent iron chelator and contributes to the anti-oxidative properties [34]. Consistent with this concept, DEXRA was reported to improve EC function in patients with hyperhomocysteinemia [35]. However, ADR-925 was not protective in a model of anthracycline-induced CVT [34]. In addition, previous work from our group demonstrated that N-acetylcysteine (an antioxidant) did not prevent EC dysfunction early after DOX treatment, again partially invalidating the antioxidant mechanisms of action [7], probably due to the acute damage present in the early phase. Taken together, it seems improbable that DEXRA (or its metabolite ADR-925) prevents EC dysfunction via scavenging of free reactive oxygen species. Alternatively, DOX-induced EC dysfunction has been associated with alterations in eNOS expression or phosphorylation, although this was not consistent between studies [36]. In our study, neither DOX nor DEXRA affected eNOS, or phosphorylated eNOS (Ser-1177) levels significantly in aortic segments, although our data may suggest a trend towards reduction of phosphorylated-eNOS in DOX-treated animals.

### DEXRA prevents DOX-induced cardiotoxicity

Our results confirm DEXRA’s cardiac protective properties [37]. The chronic DOX model showed moderate reduction of LVEF as well as increased lung weight, indicating pulmonary oedema as a sign of heart failure, which was prevented by DEXRA. Additionally, DOX-induced weight loss (probably due to skeletal muscle atrophy [38]), which remarkably was not prevented by DEXRA.

DNA damage induced by DOX is a central mechanism of its CVT, mediated by the inhibition of TOP-2β [39], which is counteracted by DEXRAs ability to degrade TOP-2β [40]. In alignment, we observed an increase in DNA damage in the myocardium of DOX-treated mice, but not in DEXRA-treated animals. Noteworthy, TOP-2β was present in both cardiac and aortic tissue, but no decline was observed in DEXRA-treated mice. Given the short half-life of DEXRA [41] our sampling interval (4–5 days after DEXRA) may be too long. Although, previously we also showed no difference in TOP-2β in the acute DOX/DEXRA setting [7].

In addition, DOX-induced cardiac injury was recently linked to ferroptosis in mice [16]. However, the dosing regimen involved intraperitoneal injection of a single high-dose DOX (10 or 20 mg/kg) [16], probably not reflecting human cardiotoxicity. Further, a study in rats using lower cumulative doses (2.5 mg/kg/week DOX for 6 weeks) also reported ferroptosis, as well as an increase of the iron exporter FERROPORTIN-1 [42]. Our data also show increased *Ferroportin-1* expression in the heart. Interestingly, DEXRA seems to limit *Ferroportin-1* overexpression in the murine heart, possibly due to the iron-chelating capabilities of the ADR-925 metabolite [14]. In aortic segments, however, *Ferroportin-1* gene expression was inconclusive due to greater variability.

### SERPINA3 upregulation prevented by DEXRA treatment

Previously, we reported the serin proteasome inhibitor SERPINA3 (mouse ortholog SERPINA3N) as a marker for DOX-related CVT in patients and mice [2, 43]. DEXRA prevented the SERPINA3N response induced by DOX in cardiac tissue and plasma, indicating the preventive properties of DEXRA against CVT. Moreover, this suggests that SERPINA3 could serve as a potential biomarker for monitoring DOX-CVT and evaluating a patient's response to DEXRA. Previously, we showed that SERPINA3(N) correlated with cancer treatment-induced reduction of LVEF in both humans and mice [2]. Delrue et al. showed that increased circulating SERPINA3 was associated with higher mortality in heart failure patients [44]. While literature still points towards the liver as a primary source of SERPINA3 in plasma [45, 46] we showed *SERPINA3* expression in LV biopsies of CVT patients. Additionally, vascular, and cardiac tissue-specific upregulation of SERPINA3N in mice was demonstrated [43]. Noteworthy, circulating levels of SERPINA3 in humans were 10,000–fold higher than in mice. Despite differences in circulating protein levels between mice and humans, our data consistently highlight upregulation of SERPINA3 in the progression of CVT, although further research is warranted to elucidate the mode of action of SERPINA3.

## Conclusion

Our study demonstrates, for the first time, that DEXRA prevents DOX-induced vascular toxicity while confirming its cardioprotective effects. DOX increased PWV, a marker of arterial stiffness, and this detrimental response was completely attenuated by DEXRA. Additionally, DEXRA fully restored EC dysfunction elicited by DOX and prevented the rise in SERPINA3 expression in aortic-, cardiac tissue, and plasma induced by DOX. These findings suggest that DEXRA mitigates chronic vascular toxicity induced by DOX, by preventing EC dysfunction and VSMC hypercontractility. Additionally, the study reinforces the potential of SERPINA3 as a biomarker for DOX-induced CVT and the protective role of DEXRA, which could guide the development of clinical diagnostics for monitoring and treating chemotherapy-related CV complications.

## Supplementary Information

Supplementary Material 1.

## Data Availability

The datasets used and/or analyzed during the current study are available from the corresponding author upon reasonable request.

## Abbreviations

CVT: Cardiovascular toxicity
DEXRA: Dexrazoxane
DEANO: Diethylamine NONOate
DOX: Doxorubicin
EC: Endothelial cell
eNOS: Endothelial nitric oxide synthase
L-NAME: Nω-nitro-L-arginine methyl ester
LVEF: Left ventricular ejection fraction
ROS: Reactive oxygen species
Topoisomerase-2β: TOP-2β
VSMC: Vascular smooth muscle cells

## Data availability

No datasets were generated or analysed during the current study.
