# Supplementary material for: Dexrazoxane prevents vascular toxicity in doxorubicin-treated mice
Source: Cardiooncology. 2024 Oct 4;10:65. doi: 10.1186/s40959-024-00270-w (PMC11451066; doi:10.1186/s40959-024-00270-w)
Supplement: Supplementary file 1 — Supplementary Material 1. [file 40959_2024_270_MOESM1_ESM.docx]

# **Supplementary Appendix**

# **Dexrazoxane prevents vascular toxicity in doxorubicin-treated mice**

**Dustin N. Krüger, MSc. ^a^, Matthias Bosman, PhD ^a^, Emeline M. Van Craenenbroeck, M.D., PhD ^b,c^, Guido R.Y. De Meyer, PhD ^a^, Constantijn Franssen, M.D., PhD. ^b,c,†^ and Pieter-Jan Guns, PhD ^a,†^**

^a^ Laboratory of Physiopharmacology, Faculty of Medicine and Health Sciences, Faculty of Pharmaceutical, Biomedical and Veterinary Sciences, Campus Drie Eiken, University of Antwerp, Universiteitsplein 1, B-2610 Antwerp, Belgium

^b^ Research Group Cardiovascular Diseases, University of Antwerp, B-2610 Antwerp, Belgium

^c^ Department of Cardiology, Antwerp University Hospital (UZA), Drie Eikenstraat 655, B-2650 Edegem, Belgium

The Laboratory of Physiopharmacology and the Research Group Cardiovascular Diseases are part of the Infla-Med Centre of Excellence of the University of Antwerp.

† Shared senior authorship.

**Corresponding author:**

Dustin Nicolas Krüger

Mailing address: University of Antwerp, Faculty of Medicine and Health Sciences, Laboratory of Physiopharmacology, Campus Drie Eiken, Universiteitsplein 1, B-2610, Antwerp, Belgium.

Tel. : +32 (0) 456 325045

E-mail: [dustin.krueger@icloud.com](mailto:dustin.krueger@icloud.com)

ORCID: https://orcid.org/0000-0003-0752-3819

# **Supplemental table**

**Supplementary Table 1: Echocardiographic evaluation of cardiac function at week 0, 2 and 6 in each group.**

| **Week 0** | DOX 0 mg/kg; DEXRA 0 mg/kg | | | |  |  |
| --- | --- | --- | --- | --- | --- | --- |
|  | **VEHICLE** | **DEXRA** | **DOX** | **DEXRA + DOX** | **2-Way ANOVA** | **Kruskal-Walis** |
| LVEF(%) | 69 ± 1 | 65 ± 1 | 69 ± 2 | 67 ± 2 | ns |  |
| LV FS (%) | 39 ± 1 | 36 ± 1 | 39 ± 1 | 35 ± 2 |  | ns |
| LVAW (mm; diastole) | 0.6 ± 0.0 | 0.5 ± 0.1 | 0.5 ± 0.0 | 0.5 ±0.0 | ns |  |
| LVAW (mm; systole) | 0.7 ± 0.0 | 0.7 ±0.0 | 0.7 ± 0.0 | 0.7 ± 0.0 | ns |  |
| LVID (mm; diastole) | 4.2 ± 0.1 | 4.5 ± 0.2 | 4.4 ± 0.1 | 4.5 ± 0.1 | ns |  |
| LVID (mm; systole) | 2.6 ± 0.1 | 2.9 ± 0.1 | 2.7 ± 0.1 | 2.9 ± 0.1 | ns |  |
| LVPW (mm; diastole) | 0.6 ± 0.1 | 0.7 ± 0.1 | 0.7 ± 0.1 | 0.6 ± 0.1 | ns |  |
| LVPW (mm; systole) | 1.2 ± 0.1 | 1.2 ± 0.1 | 1.2 ± 0.0 | 1.2 ± 0.0 | ns |  |
| SV (µL) | 55 ± 3 | 61 ± 5 | 61 ± 4 | 58 ± 2 | ns |  |
| E/A (ratio) | 1.4 ± 0.1 | 1.5 ± 0.1 | 1.5 ± 0.2 | 1.4 ±0.0 |  | ns |
| E/E' (ratio) | 38 ± 3 | 46 ± 5 | 38 ± 4 | 42 ± 4 | ns |  |
| **Week 2** | *DOX 8 mg/kg; DEXRA 80 mg/kg* | | | |  |  |
|  | **VEHICLE** | **DEXRA** | **DOX** | **DEXRA + DOX** | **2-Way ANOVA** | **Kruskal-Walis** |
| LVEF(%) | 66 ± 2 | 64 ± 1 | 55 ± 2 | 59 ± 1 | DOX ******* |  |
| LV FS (%) | 37 ± 1 | 35 ± 1 | 29 ± 1 | 31 ± 1 | DOX ******** |  |
| LVAW (mm; diastole) | 0.6 ± 0.0 | 0.6 ± 0.0 | 0.7 ± 0.0 | 0.7 ± 0.1 | ns |  |
| LVAW (mm; systole) | 0.9 ± 0.0 | 0.9 ± 0.1 | 0.9 ± 0.1 | 0.9 ± 0.0 | ns |  |
| LVID (mm; diastole) | 4.2 ± 0.1 | 4.6 ± 0.1 | 4.3 ± 0.1 | 4.5 ± 0.1 | ns |  |
| LVID (mm; systole) | 2.7 ± 0.1 | 3.0 ± 0.1 | 3.1 ± 0.1 | 3.1 ± 0.1 | DOX ***** |  |
| LVPW (mm; diastole) | 0.7 ± 0.1 | 0.7 ± 0.1 | 0.8 ± 0.0 | 0.6 ± 0.1 | ns |  |
| LVPW (mm; systole) | 1.3 ± 0.1 | 1.2 ± 0.1 | 1.2 ± 0.1 | 1.2 ± 0.1 | ns |  |
| SV (µL) | 53 ± 2 | 63 ± 5 | 45 ± 2 | 54 ± 2 | DOX ***** |  |
| E/A (ratio) | 1.3 ± 0.1 | 1.5 ± 0.1 | 1.5 ± 0.2 | 1.4 ± 0.1 |  | ns |
| E/E' (ratio) | 38 ± 3 | 38 ± 2 | 38 ± 2 | 38 ± 3 |  | ns |
| **Week 6** | *DOX 24 mg/kg; DEXRA 240 mg/kg* | | | |  |  |
|  | **VEHICLE** | **DEXRA** | **DOX** | **DEXRA + DOX** | **2-Way ANOVA** | **Kruskal-Walis** |
| LVEF (%) | 66 ± 1 | 64 ± 1 | 51 ± 1 | 62 ± 2 | DOX ∙ DEXRA ****** |  |
| LV FS (%) | 36 ± 1 | 35 ± 1 | 26 ± 1 | 32 ± 2 | DOX ∙ DEXRA ***** |  |
| LVAW (mm; diastole) | 0.6 ± 0.1 | 0.5 ± 0.0 | 0.5 ± 0.0 | 0.5 ± 0.0 |  | ns |
| LVAW (mm; systole) | 0.9 ± 0.0 | 0.9 ± 0.0 | 0.8 ± 0.0 | 0.8 ± 0.0 | DOX ******* |  |
| LVID (mm; diastole) | 4.5 ± 0.1 | 4.9 ± 0.1 | 4.2 ± 0.1 | 4.6 ± 0.1 | DOX ******* |  |
| LVID (mm; systole) | 2.9 ± 0.1 | 3.1 ± 0.1 | 3.1 ± 0.1 | 3.0 ± 0.1 |  | **^#^** |
| LVPW (mm; diastole) | 0.7 ± 0.1 | 0.6 ± 0.1 | 0.7 ± 0.1 | 0.7 ± 0.0 | ns |  |
| LVPW (mm; systole) | 1.9 ± 0.1 | 1.3 ± 0.1 | 1.0 ± 0.1 | 1.1 ± 0.0 | DOX ***** |  |
| SV (µL) | 60 ± 2. | 72 ± 6. | 41 ± 2 | 54 ± 5 |  | **^###^** |
| E/A (ratio) | 1.4 ± 0.1 | 1.6 ± 0.2 | 1.4 ± 0.2 | 1.4 ± 0.1 |  | ns |
| E/E' (ratio) | 42 ± 3 | 39 ± 2. | 33 ± 5 | 44 ± 4 |  | ns |

For each cohort, based on normal distribution either a Two-way ANOVA or a Kruskal-Walis test was performed. ANOVA: *, ***, **** p < 0.05, 0.001, 0.0001; Kruskal-Walis ^#^, ^###^ p < 0.05, 0.001. DEXRA, dexrazoxane; DOX, doxorubicin; FS, fractional shortening; LVAW, left ventricular anterior wall; LVEF, left ventricular ejection fraction; LVID, left ventricular internal diameter; LVPW, left ventricular posterior wall; ns, not significant; SV, stroke volume.

# **Supplemental figures**

**Supplementary Figure 1: Body- and organ weight measurements.** DOX and DEXRA reduced body weight of the animal with an increase in cumulative dose. **A**: Body weight measurements over the whole treatment period. **B:** Heart- to bodyweight ratio. **C:** Lung- to body weight ratio. Dosages shown, represent cumulative doses at the specific timepoint Data is shown as mean ± SEM. **A-C:** Two-Way ANOVA and a Tukey correction. For each cohort: n = 8. *, **, p < 0.05, 0.01

**Supplementary Figure 2: TOPOISOMERASE-2β protein content in the thoracic aorta and the heart at week 6.** Neither DEXRA nor DOX affected TOP-2β levels in the heart and aorta **A:** Representative western blot for TOP-2β in cardiac tissue. **B:** Quantification of TOP-2β quantification in cardiac tissue **C:** Representative western blot for TOP-2β in aortic tissue **D:** Quantification in aortic tissue **E:**TPS of blot shown in A **F:** TPS of blot shown in C. TPS was used as loading control. TOP-2β, topoisomerase-2β; TPS, Total protein stain. At week 6 a cumulative dose of 24 mg/kg DOX and 240 mg/kg DEXRA were used. Data shows mean ± SEM. B & D Kruskal-Walis test. For each cohort: n = 8

**Supplementary Figure 3: eNOS and P-eNOS in the thoracic aorta.** No effects of DOX or DEXRA on eNOS were observed. **A:** Representative western blot for eNOS and P-eNOS-P(Ser-1177) **B:** Quantification of eNOS in aortic tissue **C:** Quantification of P-eNOS (Ser-1177) in aortic tissue. **D:** TPS of blot shown in A. TPS was used as loading control. eNOS, endothelial nitric oxide synthetase; TPS, Total protein stain. At week 6 a cumulative dose of 24 mg/kg DOX and 240 mg/kg DEXRA were used. Data shows mean ± SEM. **B & C:** Kruskal-Walis test. For each cohort: n = 8

**Supplementary Figure 4: Ferroportin-1 mRNA expression in cardiac and aortic tissue.** In cardiac tissue, Ferroportin-1 was increased in DOX-treated animals, while in aortic tissue Ferroportin-1 was increased in the DEXRA and DOX-treated animals. **A:** mRNA expression in cardiac tissue. **B:** Ferroportin-1 expression in aortic tissue. Data shows mean ± SEM. **A-C:** Two-Way ANOVA and a Tukey correction. For each cohort: n = 8. *, **, p < 0.05, 0.01.
